# Antimicrobial Resistance in Bacteria Isolated From Cats and Dogs From the Iberian Peninsula

**DOI:** 10.3389/fmicb.2020.621597

**Published:** 2021-01-20

**Authors:** Yanli Li, Rubén Fernández, Inma Durán, Rafael A. Molina-López, Laila Darwich

**Affiliations:** ^1^Departament de Sanitat i Anatomia Animal, Universitat Autònoma de Barcelona, Barcelona, Spain; ^2^Departamento Veterinaria de Laboratorios Echevarne, Barcelona, Spain; ^3^Catalan Wildlife Service, Centre de Fauna Salvatge de Torreferrussa, Barcelona, Spain

**Keywords:** antimicrobial resistance, bacteria, cats, dogs, Iberian Peninsula

## Abstract

Pet animals are assumed to be potential reservoirs in transferring antimicrobial resistance (AMR) to humans due to the extensively applied broad-spectrum antimicrobial agents and their close contact with humans. In this study, microbiological data and antimicrobial susceptibility results of dog (*n* = 5,086) and cat (*n* = 789) clinical samples from a private Laboratory of Diagnosis in Barcelona were analyzed. Samples came from different counties of the Iberian Peninsula during 2016–2018. In dogs, clinical samples were most commonly from otitis, and in cats from wounds, respiratory tract infections and conjunctivitis. In both pet groups, *Staphylococcus* spp. (31% in dogs vs 30% in cats), *Streptococcus* spp. (19% vs 17%), *Pseudomonas* spp. (16% vs 10%), *Escherichia coli* (8% vs 5.6%), and *Enterococcus* spp. (5.5% vs 6.8%) were shown as the most predominant bacteria. However, higher frequencies of *P. aeruginosa*, *P. canis*, and *S. pseudintermedius* were found in dogs, while *S. aureus* and *P. multocida* were more prevalent in cats. The antimicrobial susceptibility testing demonstrated that *Enterococcus* spp. and *Pseudomonas* spp. presented the highest levels of AMR in both dogs and cats. Within the Enterobacteriaceae, *E. coli* showed low levels of AMR compared to *Klebsiella*, *Proteus*, or *Enterobacter* spp. Respiratory tract infections caused by *K. pneumoniae* presented higher AMR in cats. By contrast, *Pasteurella* isolates from the respiratory tract were highly sensitive to all the antimicrobials in cats and dogs. Data from this study could be used to guide empirical antimicrobial selection in companion animal veterinary practices in the Iberian Peninsula.

## Introduction

The emergence of antimicrobial resistance (AMR) has become a great concern worldwide, threatening the public healthcare system (Brinkac et al., 2017). Some studies assumed that food animals were the main contributors of human AMR by transferring resistant bacteria or genes through food chain (Witte, 1998; Fey et al., 2002; Smith et al., 2002; White et al., 2002; Angulo et al., 2009; McEwen and Fedorka-Cray, 2017). However, (Barber et al., 2016) established a new analytical model and assumed the non-foodborne transmission of AMR should be equally emphasized. Thus, the companion animals, mostly dogs and cats, started to be considered a potential reservoirs of AMR due to their close contact with humans and being extensively treated by broad-spectrum antimicrobial agents (Guardabassi et al., 2004; Lloyd, 2007). If AMR can be transmitted to humans from companion animals, and if multi-drug resistant (MDR) bacteria exist among the household pets, the risk of antimicrobial treatment failure would highly increase in both animals and humans. Thus, understanding the prevalence of AMR among pets, mainly dogs and cats, is demanded from both veterinary and human medicine perspectives. However, due to the clinical cases are not always entirely recorded and monitored, the available data on pet-related AMR are very minimal.

In this study, we analyzed the clinical microbiological data on pet dogs and cats with data collected between 2016 and 2018 in the Iberian Peninsula, and found out the most prevalent bacterial infections and AMR profiles among the two companion animals.

## Materials and Methods

### Data Source and Management

**FIGURE 1:**
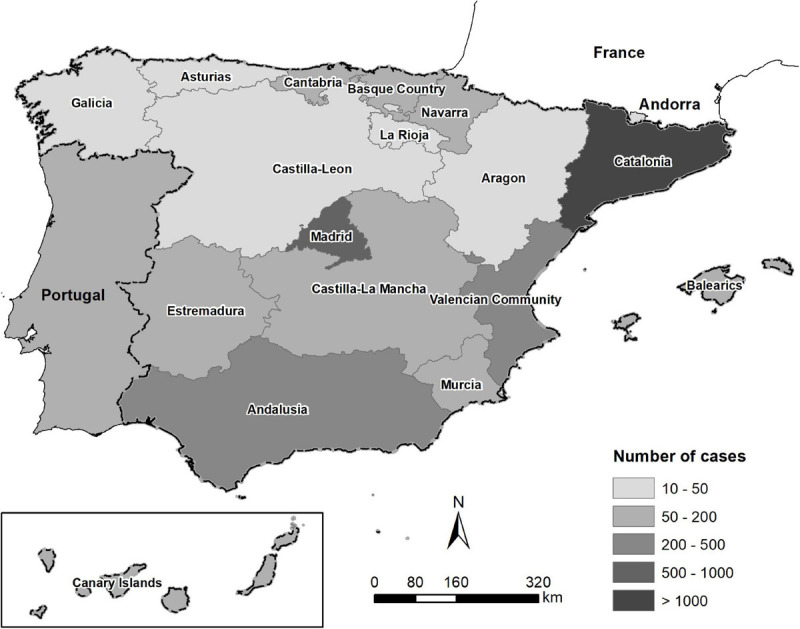
Map of the origin and the number of clinical specimens from dogs and cats in the Iberian Peninsula between 2016 and 2018.

Retrospective records of 5,875 microbiological analyses of clinical specimens from dogs (*n* = 5,086) and cats (*n* = 789) between 2016 and 2018 were analyzed in the present study. The records were provided by the Veterinary Medicine Department of a large private Laboratory of Diagnosis in Barcelona. The lab records contained information about clinical cases submitted by veterinary clinics covered throughout the Spanish provinces, Portugal, and Andorra (Figure 1). Data were assessed for duplicates and missing information. Finally, only samples with complete records were analyzed. Repeat samples of the same case were not included. The following variables were extracted from the records: animal species, type/origin of sample, county of specimen, bacterial identification, and antimicrobial susceptibility testing.

The specimens were classified according to the sample origin as follows: otitis (*n* = 3,043), wounds (*n* = 1,142), respiratory tract infections (which included rhinitis, bronchitis, pneumonia, and pleuritic, *n* = 483), dermatitis (*n* = 341), abscesses (*n* = 218), conjunctivitis (*n* = 190), and others (which included reproductive tract infections, musculoskeletal infections, arthritis, and osteomyelitis, *n* = 458). Urine samples were not included in the study.

### Microbiological Analysis and Antimicrobial Susceptibility Testing

Microbiological identification was performed using the MALDITOF mass spectrometeror the API^®^, ID system (bioMérieux, Spain). All Gram-positive bacterial isolates were performed by the antimicrobial susceptibility test using the standard disk diffusion method according to Performance Standards for Antimicrobial Susceptibility Testing for bacteria isolated from animals (M31-A3, CLSI VET01, 2008) and from humans (M100-S24, CLSI, 2016) for drugs not licensed for veterinary use. The panel included the following antimicrobial classes: beta-lactams (amoxicillin-clavulanic acid, oxacillin, cefoxitin, penicillin, piperacillin, piperacillin/tazobactam, ampicillin, cephalexin, cephalotin, cefazolin, cefuroxime, ceftazidime, cefotaxime, cefovecin, cefotaxim, and cefepime), carbapenems (imipenem and meropenem), and aztreonam; fluoroquinolones (ciprofloxacin, enrofloxacin, and marbofloxacin); aminoglycosides (amikacin, gentamicin, tobramycin, and neomycin); macrolides (azithromycin and erythromycin); tetracyclines (doxycycline); clindamycin; polymyxin B; trimethoprim/sulphamethoxazole; chloramphenicol/florphenicol; fosfomycin; mupiracin; and glycopetides (vancomycin). For Gram negative bacteria, NM44 MicroScan (Beckman Coulter, Villepinte, France) system was performed for all the antimicrobials except for those antibiotics authorized for veterinary uses that are not included in the automatic scan panels (enrofloxacin, pradofloxacin, marbofloxacin, doxycycline, cephalexin, and cefovecin). The MicroScan is an automated bacterial identification and susceptibility testing system based on microbiology principles of true minimum inhibitory concentration (MIC) testing. Based on the lab readings, isolates were classified as Susceptible, Intermediate or Resistant. For statistical assessments, isolates that exhibited intermediate resistance were re-classified as resistant. The laboratory has the quality management system certificate ISO-9001 since 1998 and the accreditation from ENAC (National Accreditation Entity) according to criteria included in the ISO/IEC 17025 Standard defined in the Technical Annexes 511/LE1947 for Pharmaceutical Toxicology and Microbiology Testing.

### Statistical Analysis

Descriptive and statistical analysis was performed using the SPSS Advanced Models TM 15.0 (SPSS Inc. 233 South Wacker Drive, 11th Floor Chicago, IL, United States 60.606-6412). The Chi-square (χ^2^) or Fishers Exact tests were used to compare bacterial spp. and the AMR frequencies in both animal groups. Statistical significant was considered when *p* < 0.05.

## Results

### Microbiological Diagnosis of Bacterial Infections

**FIGURE 2:**
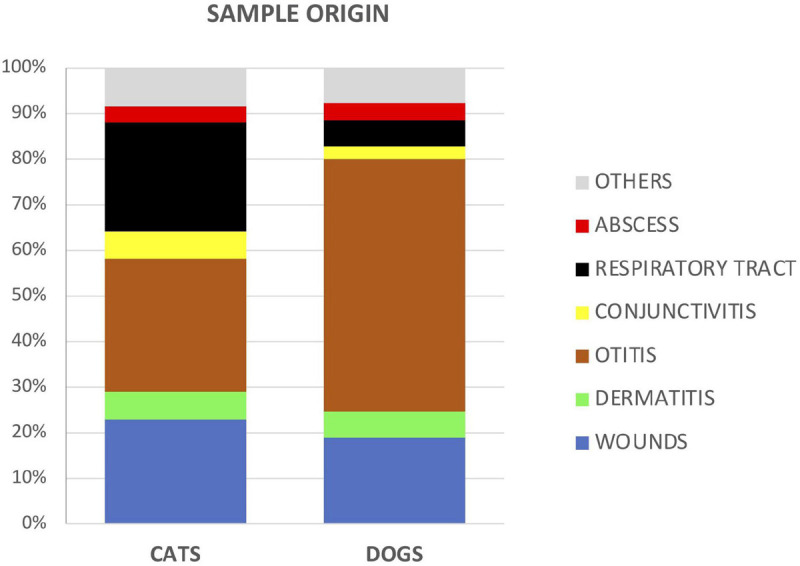
Proportion of analyzed samples between cats and dogs according to the type or source of specimens.

In dogs, most of the samples remitted to the lab were from cases related to otitis (55.3% dogs vs 29% cats, χ^2^ = 187.2, and *p* < 0.05). In cats, samples from wounds (23% cats vs 19% dogs, χ^2^ = 6.6, and *p* = 0.01), respiratory tract infections (24% vs 5.8%, χ^2^ = 299, and *p* < 0.05), and conjunctivitis (6% vs 2.8% χ^2^ = 21.6, and *p* < 0.001) were more frequently remitted (Figure 2).

*Staphylococcus* spp. (31–30%), *Streptococcus* spp. (19–17%) and *Pseudomonas* spp. (16–10%), followed by *Escherichia coli* (8.0–5.6%), and *Enterococcus* spp. (5.5–6.8%), were the most predominant bacteria isolated in both dogs and cats (Table 1). As a differential trait, dogs presented higher frequencies of *Pseudomonas aeruginosa* (92% vs 72%), *P. canis* (36.7% vs 6.5%), and *S. pseudintermedius* (17% vs 4.6%), while *S. aureus* (6% vs 1.5%) and *P. multocida* (63% vs 20.4%) were more prevalent in cats (Table 1).

**TABLE 1:** Frequencies of bacterial species identified in dog and cat specimens.

		**DOGS (*N* = 5,086)**		**CATS (*N* = 789)**
		***n* (%)**		***n* (%)**

*Acineto bacter* spp.		61 (1)		18 (2)
	*A. baumannii*	22 (36.1)	*A. lwoffii*	8 (44.4)
	*A. lwoffii*	14 (23)	*A. baumannii*	2 (11.1)
	*A. haemolyticus*	4 (6.6)	*A. haemolyticus*	1 (5.5)
	Others	2 (3.3)		
*Bordetella* spp.		47 (0.9)		15 (1.9)
	*B. bronchi septica*	47 (100)	*B. bronchiseptica*	15 (100)
*Candida* spp.		30 (0.5)		7 (0.9)
	*C. parapsilosis*	5 (16.7)	*C. parapsilosis*	3 (42.9)
	*C. albicans*	2 (6.5)	*C. albicans*	2 (28.6)
	Others	4 (13.3)		
*Coryne bacterium* spp.		194 (3.8)		22 (2.8)
	*C. amycolatum*	7 (3.6)	*C. amycolatum*	2 (9.1)
	*C. auriscanis*	5 (2.6)		
	Others	2 (1)		
*Entero bacter* spp.		84 (1.6)		26 (3.3)
	*E. cloacae*	59 (70.2)	*E. cloacae*	22 (84.6)
	*E. aerogenes*	13 (15.5)	*E. aerogenes*	3 (11.5)
	*E. gergoviae*	8 (9.5)	*E. gergoviae*	1 (3.8)
*Entero coccus* spp.		281 (5.5)		54 (6.8)
	*E. faecalis*	92 (32.7)	*E. faecalis*	18 (69.2)
	*E. faecium*	8 (2.8)	*E. avium*	2 (7.7)
	*E. canintestini*	1 (0.4)	*E. faecium*	1 (3.8)
	*E. durans*	1 (0.4)	*E. hirae*	1 (3.8)
*Escherichia* spp.		405 (8)		44 (5.6)
	*E. coli*	400 (98.8)	*E. coli*	42 (95.5)
	*E. vulneris*	4 (1)		
*Klebsiella* spp.		103 (2)		23 (2.9)
	*K. pneumoniae*	73 (70.9)	*K. pneumoniae*	17 (73.9)
	*K. oxytoca*	28 (27.2)	*K. oxytoca*	6 (26.1)
	*K. ornithinolytica*	1 (1)		
*Pasteurella* spp.		49 (1)		62 (7.8)
	*P. canis*	18 (36.7)	*P. multocida*	39 (62.9)
	*P. multocida*	10 (20.4)	*P. canis*	4 (6.5)
	*P. pneumotropica*	3 (6.1)	Others	4 (6.5)
*Proteus* spp.		205 (4)		5 (0.6)
	*P. mirabilis*	198 (96.6)	*P. mirabilis*	5 (100)
	*P. vulgaris*	3 (1.5)		
*Pseudo monas* spp.		827 (16.3)		76 (9.6)
	*P. aeruginosa*	761 (92)	*P. aeruginosa*	55 (72.4)
	*P. fluorescens*	18 (2.2)	*P. fluorescens*	5 (6.6)
	Others	34 (4.1)	Others	16 (21.1)
*Serratia* spp.		104 (2)		14 (1.7)
	*S. marcescens*	96 (92.3)	*S. marcescens*	12 (85.7)
	*S. liquefaciens*	7 (6.7)	*S. liquefaciens*	2 (14.3)
*Staphylo coccus* spp.		1,581 (31)		239 (30.3)
	*S. pseudin termedius*	275 (17.4)	*S. aureus*	14 (5.9)
	*S. intermedius*	109 (6.9)	*S. epidermidis*	12 (5)
	*S. schleiferi*	30 (1.9)	*S. felis*	12 (5)
	*S. aureus*	23 (1.5)	*S. pseudintermedius*	11 (4.6)
	*S. epidermidis*	9 (0.6)	*S. schleiferi*	2 (0.8)
	Others	25 (1.6)	Others	16 (6.7)
*Strepto coccus* spp.		972 (19)		132 (16.7)
	*S. canis*	23 (2.4)	*S. canis*	2 (1.5)
	*S. dysgalacticae*	3 (0.3)		
	*S. halichoeri*	1 (0.1)		

**FIGURE 3:**
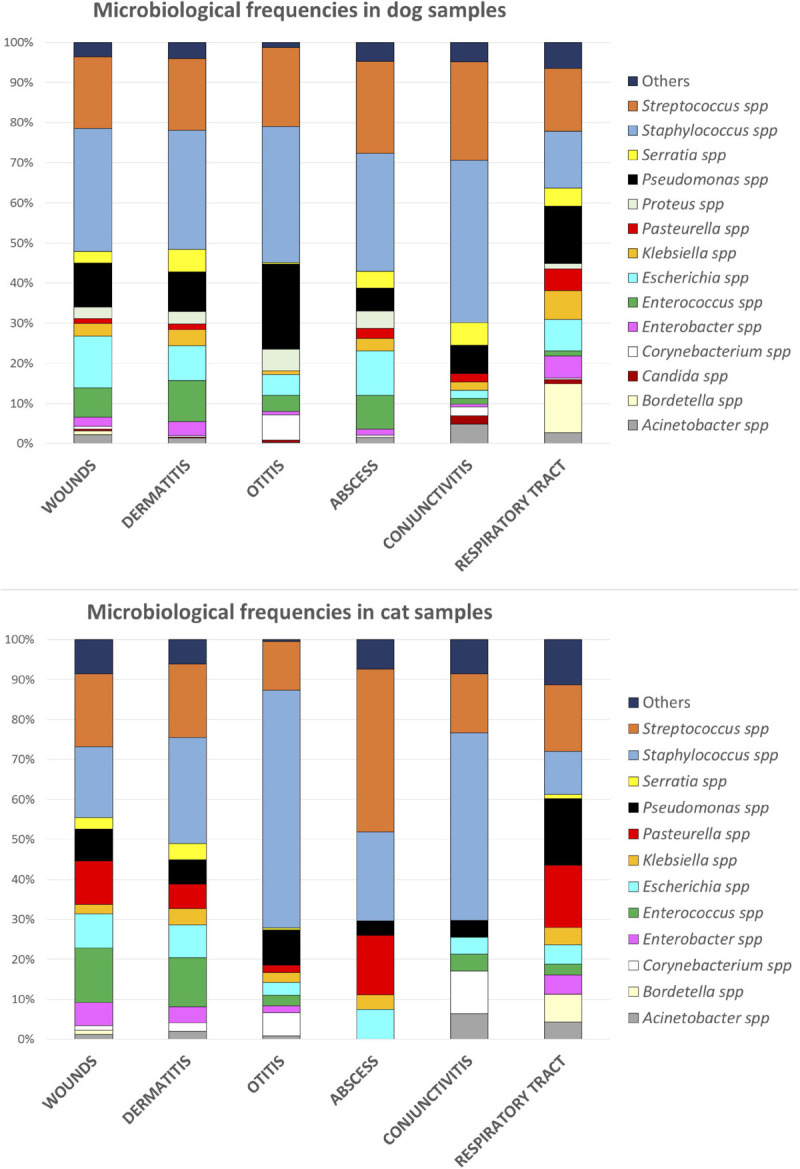
Frequencies of bacterial species according to the origin of infections in dogs and cats.

The distribution of pathogens for different sample categories showed that wounds and dermatitis presented similar patterns of distribution in dogs and cats, with *Staphylococcus, Streptococcus, Enterococcus*, and *E. coli* identified as the most frequently isolated agents (Figure 3). From otitis specimens, infections by *Staphylococcus* spp. were highly detected in both cats and dogs; meanwhile in cats, high frequencies of *P. aeruginosa* and *E. coli* were presented. On the other hand, dogs presented in general a larger bacterial diversity in samples from abscess, conjunctivitis and respiratory tract infections in comparison to cats. In this line, cats showed higher percentages of *Bordetella* spp. and *P. multocida* infections in conjunctivitis and respiratory specimens, respectively, (Figure 3).

### Antimicrobial Susceptibility Testing

Comparisons of AMR levels between dogs and cats were only made for bacterial species, which were recorded for more than 20 different strains in the antibiotic sensitivity test. Thus, the following species were involved: *Staphylococcus* spp. (*n* = 1,572 isolates from dogs, *n* = 239 from cats), *Streptococcus* spp. (*n* = 969, *n* = 132), *Enterococcus* spp. (*n* = 281, *n* = 54), *Escherichia* spp. (*n* = 405, *n* = 44), *Enterobacter* spp. (*n* = 193, *n* = 22), *Klebsiella* spp. (*n* = 103, *n* = 23), *Pseudomonas* spp. (*n* = 825, *n* = 76), *Pasteurella* spp. (*n* = 49, *n* = 62), and *Corynebacterium* spp. (*n* = 194, *n* = 22). In addition, for the most relevant gram-negative bacteria species, minimal inhibitory concentration (CMI) values required to inhibit the growth of 50% (MIC50) and 90% (MIC90) of organisms were assessed for some antimicrobials (Table 2). Interestingly, the Enterobacteriaceae species presented high values of CMI90 for beta-lactams, ciprofloxacin, gentamicin and trimethoprim/sulfamethoxazole in both animal groups. *Pseudomonas* spp. showed the highest CMI50 values for amoxicillin-clavulanate and cefoxitin (jointly with *Enterobacter* spp.), for ampicillin (jointly with *Klebsiella* spp.), and for cefotaxime and cefuroxime. Finally, *Proteus* spp. isolated from dogs presented a CMI90 value = 8 mg/L to imipenem, exceeding the resistant breakpoint (Table 2).

**TABLE 2:** Minimal Inhibitory Concentrations (MIC, mg/L) values in Gram-negative bacteria isolated from dogs and cats.

	**DOG SPECIMENS**											
	***Acinetobacter* spp.**		***Pseudomonas* spp.**		***Escherichia* spp.**		***Klebsiella* spp.**		***Enterobacter* spp.**		***Proteus* spp.**	
	**MIC_50_**	**MIC_90_**	**MIC_50_**	**MIC_90_**	**MIC_50_**	**MIC_90_**	**MIC_50_**	**MIC_90_**	**MIC_50_**	**MIC_90_**	**MIC_50_**	**MIC_90_**
AMC	4	>32	>32	>32	4	>32	4	>32	>32	>32	<2	16
AMK	<2	16	>2	16	<2	<8	<2	16	<2	<8	<2	4
AMP	16	>32	>32	>32	16	>32	>32	>32	16	>32	<2	>32
CAZ	4	16	4	8	<1	16	<1	16	<1	>64	<1	<1
CIP	<0.25	>4	<0.25	>4	<0.25	>4	<0.25	>4	<0.25	>4	<0.25	>4
CTX	8	32	16	>64	<1	8	<1	>64	<1	>64	<1	4
CXM	32	>64	>64	>64	4	>64	4	>64	4	>64	<1	16
FOX	>64	>64	>64	>64	<4	>64	>8	>64	>64	>64	<4	16
GEN	<1	8	<1	8	<1	>16	<1	>16	<1	8	<1	>16
IPM	<0.25	1	2	2	<0.25	<0.5	<0.25	<0.25	<0.5	2	2	8
SXT	<20	>320	160	>320	<20	>320	<20	>320	<20	>320	<20	>320
TZP	8	16	8	32	<4	8	<4	>128	<8	>128	<4	<4

		**CAT SPECIMENS**										

	***Acinetobacter* spp.**		***Pseudomonas* spp.**		***Escherichia* spp.**		***Klebsiella* spp.**		***Enterobacter* spp.**		***Proteus* spp.**	
	**MIC_50_**	**MIC_90_**	**MIC_50_**	**MIC_90_**	**MIC_50_**	**MIC_90_**	**MIC_50_**	**MIC_90_**	**MIC_50_**	**MIC_90_**	**MIC_50_**	**MIC_90_**
	
AMC	4	16	>32	>32	4	>32	16	>32	>32	>32	8	8
AMK	<2	<8	<2	16	<2	8	<2	16	<2	16	<2	<2
AMP	4	>32	>32	>32	>16	>32	>32	>32	>32	>32	>32	>32
CAZ	4	4	16	>64	<1	>16	<1	>16	<1	>64	<1	<1
CIP	<0.25	<0.5	<0.5	>4	<0.25	>4	>2	>4	<0.25	>4	<0.25	2
CTX	8	16	16	>64	<1	>64	<1	>64	<1	>64	<1	<1
CXM	16	>64	>64	>64	4	>64	>16	>64	16	>64	<1	4
FOX	>64	>64	>64	>64	>4	8			>64	>64	<4	16
GEN	<1	<2	<1	8	<1	<2	<1	>16	<1	8	<1	>16
IPM	<0.25	<1	2	2	<0.25	<1	<0.25	<1	<0.25	<1	–	–
SXT	<20	<20	>320	>320	<20	>320	>320	>320	<20	>320	>320	>320
TZP	<8	16	8	>128	<4	64	16	>128	<8	>64	<4	<4

*AMC, Amoxicillin-clavulanic; AMK, amikacin; AMP, ampicillin; CAZ, ceftazidime; CIP, ciprofloxacin; CTX, cefotaxime; CXM, cefuroxime; FOX, cefoxitin; GEN, gentamicin; IPM, imipenem; SXT, trimethoprim/sulphamethoxazole; and TZP, piperacillin/tazobactam. CLSI (M100-S24): AMC ≥ 32/16, AMK ≥ 64, and AMP ≥ 16; CAZ ≥ 16, CIP ≥ 1, CTX ≥ 4, CXM ≥ 32, FOX ≥ 32, GEN ≥ 16, IPM ≥ 4, SXT ≥ 4/76, and TZP ≥ 128/4. CLSI (VET01): AMC ≥ 1, AMP > 8, AMK ≥ 16, CAZ ≥ 16, and GEN ≥ 8.*

**FIGURE 4:**
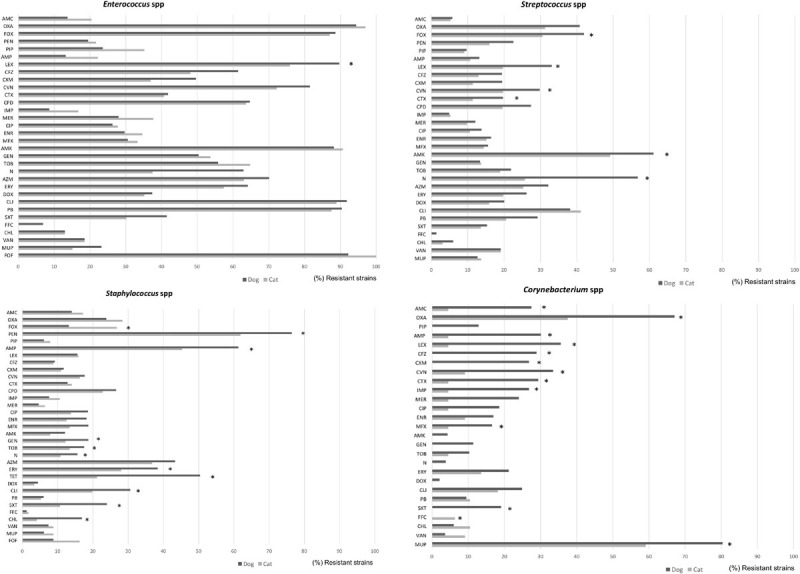
Comparison of antimicrobial resistance frequencies in Gram negative bacteria isolated from dogs and cats. Statistical significance was calculated by Chi-square (χ^2^) or Fishers Exact tests, *p < 0.05. AMC, Amoxicillin-clavulanic; AMP, ampicillin; FOX, cefoxitin; LEX, cephalexin; CFZ, cefazolin; CEF, cephalotin; CXM, cefuroxime; CAZ, ceftazidime; CTX, cefotaxime; CVN, cefovecin; FEP, cefepime; IPM, imipenem; MEM, meropenem; CIP, ciprofloxacin; ENR, enrofloxacin; MFX, marbofloxacin; PRA, pradofloxacin; AMK, amikacin; GEN, gentamicin; TOB, tobramycin; DOX, doxycycline; FOF, Fosfomycin; NIT, nitrofurantoin; and SXT, trimethoprim/sulfamethoxazole.

Among the Gram-positive bacteria, more than 80% of *Enterococcus* isolates presented resistance to oxacillin, cefoxitin, amikacin, clindamycin, polymyxin B, and fosfomycin from both dogs and cats (Figure 4). Similar patterns but with lower frequencies were detected for S*taphylococcus, Streptococcus*, and *Corynebacterium* spp., principally in isolates from dog specimens. Besides, S*taphylococcus* spp. isolated from dogs presented higher levels of AMR to macrolides, tetracycline, trimethoprim/sulfamethoxazole and chloramphenicol compared to cat isolates. Of note, a significant higher frequency of imipenem and marbofloxacin *Corynebacterium* resistant isolates were found in dog cases (Figure 4).

**FIGURE 5:**
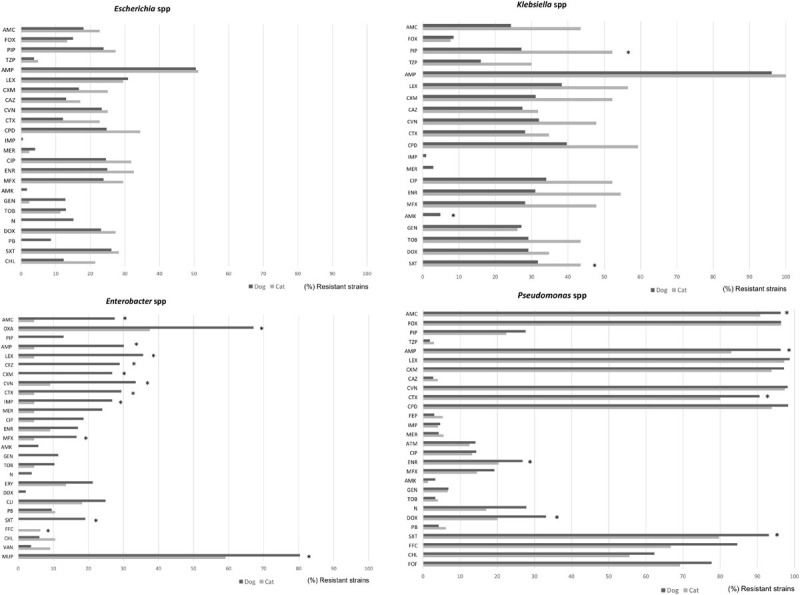
Comparison of antimicrobial resistance frequencies in Gram positive bacteria isolated from dogs and cats. Statistical significance was calculated Chi-square (χ^2^) or Fishers Exact tests, *p < 0.05. AMC, Amoxicillin-clavulanic; AMP, ampicillin; LEX, cephalexin; CFZ, cefazolin; CXM, cefuroxime; CTX, cefotaxime; CVN, cefovecin; IPM, imipenem; MEM, meropenem; CIP, ciprofloxacin; ENR, enrofloxacin; MFX, marbofloxacin; PRA, pradofloxacin; AMK, amikacin; GEN, gentamicin; TOB, tobramycin; DOX, doxycycline; ERY, erythromycin; FOF, fosfomycin; NIT, nitrofurantoin; SXT, trimethoprim/sulfamethoxazole; and VAN, vancomycin.

Within the Enterobacteriaceae family, although *E. coli* was highly isolated from wounds, dermatitis, abscesses, and otitis in both dogs and cats, they presented low levels of AMR (with the exception of ampicillin where 50% of isolates were resistant), in comparison to other members of the family such as *Klebsiella*, *Proteus*, or *Enterobacter* spp. (Figure 5). More in detail, *Enterobacter* strains from dog specimens showed a higher level of AMR to β-lactams, imipenem, and mupirocin than cats. *K. pneumoniae* from cat respiratory tract infections presented an overall higher resistance to antimicrobials than from dogs, showing statistical differences for piperacillin and trimethoprim/sulfamethoxazole (Figure 5).

Finally, *Pseudomonas* spp. presented the highest levels of AMR in both dogs and cats, showing between 80 and 97% of resistance to penicillin and cephalosporin classes, including 3rd GC, 79–94% trimethoprim/sulfamethoxazole, 68–85% flophenicol, 55–62% chloramphenicol, and 69–78% fosfomycin. In general, isolates from dogs presented higher levels of resistance than the cat isolates (Figure 5).

**FIGURE 6:**
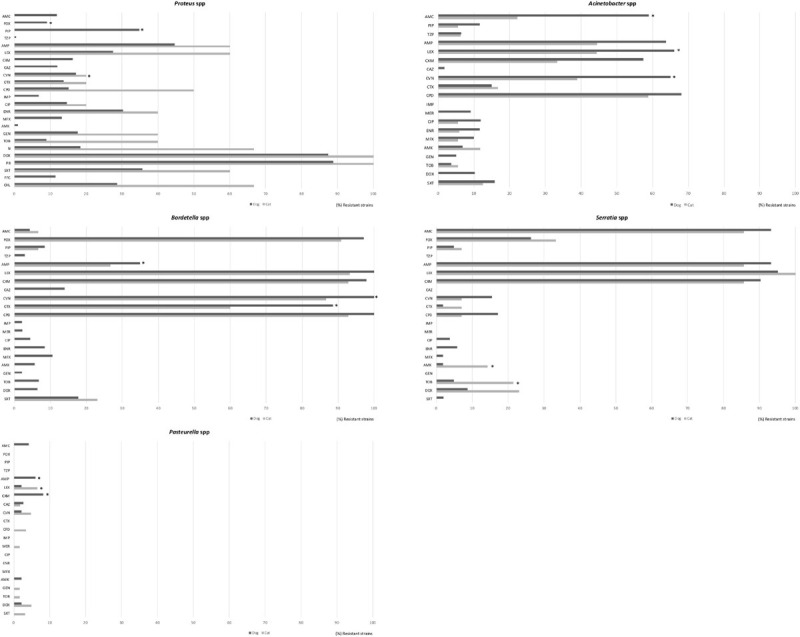
Comparison of antimicrobial resistance frequencies in other bacteria spp. isolated from dogs and less representative from cats. Statistical significance was calculated by Chi-square (χ2) or Fishers Exact tests, *p < 0.05. AMC, Amoxicillin-clavulanic; FOX, cefoxitin; PIP, piperacillin; TZP, piperacillin/tazobactam; AMP, ampicillin; LEX, cephalexin; CXM, cefuroxime; CAZ, ceftazidime; CVN, cefovecin; CTX, cefotaxime; CPD, cefpodoxime; IPM, imipenem; CIP, ciprofloxacin; ENR, enrofloxacin; MFX, marbofloxacin; AMK, amikacin; GEN, gentamicin; TOB, tobramycin; N, Neomycin; DOX, doxycycline; PB, polymyxin B, SXT, trimethoprim/sulfamethoxazole; FFC, florfenicol, and CHL, chloramphenicol.

Antimicrobial susceptibility in *Proteus* spp. (*n* = 205, *n* = 5), *Serratia* spp. (*n* = 104, *n* = 14), *Acinetobacter* spp. (*n* = 61, *n* = 18), and *Bordetella* spp. (*n* = 47, *n* = 15) was mainly done from dog isolates. (Figure 6) Interestingly, more than 80% of *Proteus* isolates were resistant to doxycycline and polymyxin B. *Acinetobacter* isolates presented a high resistance rate to cephalexin (66.1% of dog, 44.4% of cat, and *p* < 0.05), cefovecin (65.0%, 38.9%, and *p* < 0.05), ampicillin (63.8%, 44.4%), amoxicillin (59.0%, 22.2%, and *p* < 0.05), and cefuroxime (57.4%, 33.3%). Meanwhile, resistance to piperacillin, piperacilina/tazobactam, cefotaxime, ciprofloxacin, enrofloxacin, marbofloxacin, amikacin, tobramycin, and trimethoprim/sulfamethoxazole was also found in both pet groups but in a low proportion of isolates (<20%; Figure 6).

As regard *Pasteurella* isolates, they were detected principally from respiratory tract, most of the isolates were highly sensitive to all the antimicrobials in cats and dogs, showing low resistance frequencies only to cefuroxime (8.2%) and ampicillin (6.1%) in dogs, and cephalexin (6.5%) and cefovecin (4.8%) in cats (Figure 6).

## Discussion

This study provides data of the most frequently isolated bacteria from cat and dog infections and their associated AMR profiles based on a large number of clinical cases (*N* = 5875) within the Iberian Peninsula. This information can be a guide to clinicians, especially those working in this region, to make rational decisions on the use of antimicrobials, principally when empirical antimicrobial treatment is recurrent in companion animal veterinary medicine.

Most of the specimens submitted to the lab were from ears in both cats and dogs, and in cats, a large number of samples were also from respiratory tract infections and wounds. The distribution of pathogens showed that *Staphylococcus, Streptococcus, Pseudomonas, E. coli*, and *Enterococcus* were the most frequently isolated agents for different sample categories. In both cats and dogs, *Staphylococcus* spp. was commonly isolated from several sample sources including ears, skin, eyes, abscesses and wounds. This finding agrees with other studies conducted in Canada, Sweden, and South Africa (Windahl et al., 2015; Qekwana et al., 2017; Awosile et al., 2018) which confirms *Staphylococcus* spp. as an opportunistic pathogen of the integument and mucosae, causing otitis externa, pyoderma, and post-surgical complications in dogs.

Thirty-one and thirty percent of the studied samples were tested positive for *Staphylococcus* spp., respectively, in dogs and cats. In dogs, the identified species of *Staphylococcus* included *S. pseudintermedius* (17.4%), *S. intermedius* (7%), *S. schleiferi* (2%), *S. aureus* (1.5%), and *S. epidermidis* (0.6%), which presented a similar prevalence patterns as other studies reported in South Africa (Qekwana et al., 2017). The lower prevalence of *S. aureus* compared with *S. pseudintermedius* was in accordance with previously published works (Hanselman et al., 2009; Kawakami et al., 2010; Chanchaithong et al., 2014; Dos Santos et al., 2016). In cats, *S. aureus* was the most common isolated specie. The high rate of colonization with *S. pseudintermedius* and *S. aureus* found in dog and cat specimens could represent a public health concern, as has been described in many papers the potential transmission of *Staphylococcus* spp. from dogs to humans when exposing to carrier or infected dogs (Boost et al., 2007; Faires et al., 2009; Frank et al., 2009; Pantosti, 2012; Dos Santos et al., 2016).

The most common ear pathogens isolated from dogs are coagulase-positive staphylococci (*Staphylococcus pseudintermedius*) and *P. aeruginosa* (Cole et al., 1998). By contrast, a recent study conducted in France showed that the major causative agents of dog otitis were coagulase-positive staphylococci, *P. aeruginosa*, *P. mirabilis*, and streptococci (Bourély et al., 2019). In that study, the authors found that since 2003 resistance to fluoroquinolones has been decreased in both *P. aeruginosa* and *S. pseudintermedius* isolates, resulting for *P. aeruginosa*, 19.4% of isolates were resistant to both enrofloxacin and gentamicin (Bourély et al., 2019). In the present study, *S. pseudintermedius, P. aeruginosa*, and *E. coli.* were also frequently isolated from dog otitis specimens, and similar percentages of fluoroquinolones and gentamicin resistance were observed for *S. pseudintermedius* and *P. aeruginosa* isolates (<20%). Meanwhile, the *P. aeruginosa* isolates showed high levels of resistance to penicillin and cephalosporin classes (including 3rd GC), trimethoprim/sulfamethoxazole, phenicoles, and fosfomycin, both in dogs and cats. *Pseudomonas* spp. were intrinsically resistant to beta-lactams, combinations with β-lactamase inhibitors, chloramphenicol, erythromycin, and trimethoprim/sulfamethoxazole. In this study, high proportions of *Pseudomonas* were susceptible to the aminoglycosides (>95%). As well, the frequency of enrofloxacin resistance was low (27% in dogs and 20% in cats) compared to other studies conducted in Canada (Awosile et al., 2018). Enrofloxacin is commonly used systemically with concurrent topical treatment in cases of canine otitis caused by *P. aeruginosa* (Hariharan et al., 2006). These results suggest that aminoglycosides and fluoroquinolones have potential to be used as anti-pseudomonal drugs (Dowling, 1996). Our findings are also consistent with similar retrospective studies from Denmark, United States, and Canada (Petersen et al., 2002; Authier et al., 2006; Pedersen et al., 2007).

Enterococci are MDR from both intrinsic and acquired features. Specifically, *Enterococcus* spp. are naturally resistant to clindamycin, as well as to penicillin G and cephalothin, giving them a characteristic of AMR profile (Prescott et al., 2002; Delgado et al., 2007). Enterococci isolates of this study were principally isolated from wounds and dermatitis of companion animal specimens. More than 80% of *Enterococcus* isolates showed resistance to cephalosporins, clindamycin and polymyxin B, and more than 50% of them were also resistant to aminoglycosides. These results are consistent with findings from Canada, United States, Portugal (Delgado et al., 2007; Jackson et al., 2009; Awosile et al., 2018), and Spain (unpublished data) where enterococcal isolates from urinary infections had similar levels of resistance to cephalosporins, clindamycin, and polymyxin B, but high levels of susceptibility to penicillin, ampicillin, and amoxicillin-clavulanate. Thus, oral ampicillin or amoxicillin which is commonly prescribed as a first line treatment for empirical therapy in enterococcal infections could be appropriate for the studied region. Nevertheless, the increased AMR to gentamicin observed in this study could compromise the effectivity of combined therapies with ampicillin or amoxicillin (Arias et al., 2010).

In this study, *Streptococcus* spp. were highly susceptible to several antimicrobials, including penicillin, ampicillin, amoxicillin-clavulanate, trimethoprim/sulfamethoxazole, fluoroquinolones, and allowing for several likely effective choices for empirical therapy. Similar susceptibility pattern of *Streptococcus* spp. has also been reported (Pedersen et al., 2007; Awosile et al., 2018). Nevertheless, our isolates from dogs presented the highest resistance percentage for amikacin and neomycin (>50%); this finding could compromise the bactericidal activity of therapies holding aminoglycosides for the empirical treatment of streptococcal infections in dogs of the studied region.

Among the Enterobacteriaceae family, *E. coli* and *Proteus* spp. were highly isolated from wounds, dermatitis, abscesses and otitis in dog specimens in this study. The reduced susceptibility patterns of these bacterial species was found to cephalosporins (1st generation, 30% for cephalexin) and to ampicillin (50%). *Proteus* isolates presented resistance to doxycycline and polymyxin B (>80%) as well. Ampicillin was used in the susceptibility test to predict activity of amoxicillin (Weese et al., 2019), and is a good first-line option for the treatment of sporadic bacterial cystitis associated to *E. coli* in cats and dogs (Weese et al., 2011, 2019). The use of this antimicrobial for empirical treatment of *E. coli* infections should be with caution due to the rapid development of resistance caused by beta-lactamase production (Boehmer et al., 2018). Nonetheless, our results support than other antimicrobials, also effective against *E. coli* and *Proteus* spp., such as amoxicillin-clavulanate, amikacin, and gentamicin could be included as empirical selection (Awosile et al., 2018).

In the present study, *E. coli* strains isolated from dogs and cats showed low levels of AMR (with the exception of ampicillin) in comparison with other members within the same family, i.e., *Klebsiella*, *Proteus*, or *Enterobacter* spp. Accordingly, *Enterobacter* strains from dog specimens showed higher levels of AMR for β-lactams, imipenem and mupirocin compared to cats. Moreover, *K. pneumoniae* from respiratory tract infections in cats presented in general higher resistance to antimicrobials than dog specimens, mainly for piperacillin, and trimethoprim/sulfamethoxazole.

On the other hand, high susceptibility to many antimicrobials has been observed for *Pasteurella* isolates from respiratory tract of cats and dogs. This is consistent with findings in other reports (Pedersen et al., 2007; Kroemer et al., 2014; Awosile et al., 2018). Clinically, doxycycline and amoxicillin-clavulanate are often used for the treatment of Pasteurella infections (Lappin et al., 2017). Since most of the isolates were highly sensitive to antimicrobials including fluoroquinolones, aminoglycosides, and trimethoprim/sulfamethoxazole which are reasoned to be used for the treatment of Pasteurella infections in cats and dogs.

The antimicrobial options for empirical therapy can be compromised in companion animals (Prescott et al., 2002; Jung et al., 2020) basically due to: (1) the increased incidence in the last years of antimicrobial-resistant bacteria such as MDR *Enterococcus* spp., *Enterobacter* spp., *P. aeruginosa* and *K. pneumoniae*, and (2) the extended AMR to other antimicrobial families (i.e., aminoglycosides, fluoroquinolones, and carbapenems). Of note, the results obtained from pets of this study are similar to those reported in human hospitals in Spain (ESTUDIO EPINE-EPPS, 2017). The most prevalent bacterial species found in human nosocomial and community infections are *E. coli* (19.5%), *S. aureus* (9%) and *P. aeruginosa* (8%), followed by *K. pneumoniae* (6.3%), *Enterococcus* spp. (5.8%), *P. mirabilis* (3.2%), and *Enterobacter* spp. (2.2%). Moreover, CMI90 results of Enterobacteriaceae isolated from dogs and cats of this study presented values for amoxicillin-clavulanate >32–16 mg/L, ceftazidime = 8 mg/L, cefotaxime = 4 mg/L, cefuroxime > 64 mg/L, cefoxitin > 32 mg/L, and piperacillin/tazobactam = 16–4 mg/L, which have been associated with a BLEE phenotype in *E. coli*, *K. pneumoniae*, and *E. cloacae* from human isolates (Canton, 2010). Finally, the presence of *Proteus* isolates from dogs with imipenem CMI90 > 4 mg/L is highly suspicious for carbapenemasa production. To prevent the selection of BLEEs and carbapenem- resistance profiles in both human and animal medicine, is very important to implement the One Health approach, and monitor the resistance patterns of these pathogenic bacteria in companion animals (ESTUDIO EPINE-EPPS, 2017; Nigg et al., 2019; Jung et al., 2020).

Some limitations have to be considered in the present study. Firstly, data on clinical history and antimicrobial usage were not available. Secondly, some cases might have been treated empirically prior to culture and susceptibility testing. Thirdly, the use of laboratory data may represent a bias toward resistance, since cultures from complicated cases tend to be requested more often than uncomplicated cases. Finally, isolates that exhibited intermediate resistance were classified as resistant, this could have biased the results to some extent toward overestimating the resistance levels among the tested strains.

Despite these limitations, the results of this study provides information on susceptibility patterns in major cat and dog bacterial isolates from the Iberian Peninsula. These results show *Staphylococcus* spp., *Streptococcus* spp., *Pseudomonas* spp., *E. coli*, and *Enterococcus* spp. as the most predominant bacteria in cats and dogs, and with the highest levels of AMR in *Enterococcus* spp. and *Pseudomonas* spp. Within the Enterobacteriaceae, *E. coli* presented low levels of AMR compared to *Klebsiella*, *Proteus* or *Enterobacter* spp. Since dogs and cats are supposed to act as reservoirs of AMR genes that may transfer to humans, data from this study combined with clinical judgment can be used as a guide for rationalizing antimicrobial treatment of companion animals, at least in the Iberian Peninsula. Finally, optimizing antimicrobial use in the vet clinics will benefit to limit the selection and spreading of resistant bacteria not only among our pets but also among the human population.

## Data Availability Statement

The original contributions presented in the study are included in the article/supplementary material, further inquiries can be directed to the corresponding author/s.

## Ethics Statement

Authors declare no Institutional Animal Care and Use Committee (IACUC) or other approval declaration was needed.

## Author Contributions

YL and LD contributed to the analysis and the interpretation of the data and the writing of the manuscript. ID contributed to data collection. RF and RM-L contributed to data analysis. LD supervised the work. All authors contributed to the article and approved the submitted version.

## Conflict of Interest

The authors declare that the research was conducted in the absence of any commercial or financial relationships that could be construed as a potential conflict of interest.

## References

[B1] Angulo F. J. Johnson K. R. Tauxe R. V. Cohen M. L. (2009). Origins and consequences of antimicrobial-resistant nontyphoidal *Salmonella*: implications for the use of fluoroquinolones in food animals. *Microb. Drug Resist.* 6 77–83. 10.1089/mdr.2000.6.77 10868811

[B2] Arias C. A. Contreras G. A. Murray B. E. (2010). Management of multidrug-resistant enterococcal infections. *Clin. Microbiol. Infect.* 16 555–562. 10.1111/j.1469-0691.2010.03214.x 20569266 PMC3686902

[B3] Authier S. Paquette D. Labrecque O. Messier S. (2006). Comparison of susceptibility to antimicrobials of bacterial isolates from companion animals in a veterinary diagnostic laboratory in Canada between 2 time points 10 years apart. *Can. Vet. J.* 47 774–778. 16933555 PMC1524832

[B4] Awosile B. B. Mcclure J. T. Saab M. E. Heider L. C. (2018). Antimicrobial resistance in bacteria isolated from cats and dogs from the Atlantic Provinces, Canada from 1994-2013. *Can. Vet. J.* 59 885–893. 30104781 PMC6049328

[B5] Barber D. A. Miller G. Y. McNamara P. E. (2016). Models of antimicrobial resistance and foodborne illness: examining assumptions and practical applications. *J. Food Prot.* 66 700–709. 10.4315/0362-028x-66.4.700 12696700

[B6] Boehmer T. Vogler A. J. Thomas A. Sauer S. Hergenroether M. Straubinger R. K. (2018). Phenotypic characterization and whole genome analysis of extended-spectrum betalactamase-producing bacteria isolated from dogs in Germany. *PLoS One* 13:e0206252. 10.1371/journal.pone.0206252 30365516 PMC6203360

[B7] Boost M. V. O’Donoghue M. M. Siu K. H. G. (2007). Characterisation of methicillin-resistant *Staphylococcus aureus* isolates from dogs and their owners. *Clin. Microbiol. Infect.* 13 731–733. 10.1111/j.1469-0691.2007.01737.x 17484762

[B8] Bourély C. Cazeau G. Jarrige N. Leblond A. Madec J. Y. Haenni M. (2019). Antimicrobial resistance patterns of bacteria isolated from dogs with otitis. *Epidemiol. Infect.* 147 e121. 10.1017/S0950268818003278 30868979 PMC6518499

[B9] Brinkac L. Voorhies A. Gomez A. Nelson K. E. (2017). The threat of antimicrobial resistance on the human microbiome. *Microb. Ecol.* 74 1001–1008. 10.1007/s00248-017-0985-z 28492988 PMC5654679

[B10] Canton R. (2010). Interpretive reading of the antibiogram: a clinical necessity. *Enferm. Infecc. Microbiol. Clin.* 28 375–385. 10.1016/j.eimc.2010.01.001 20381926

[B11] Chanchaithong P. Perreten V. Schwendener S. Tribuddharat C. Chongthaleong A. Niyomtham W. (2014). Strain typing and antimicrobial susceptibility of methicillin-resistant coagulase-positive *Staphylococcal* species in dogs and people associated with dogs in Thailand. *J. Appl. Microbiol.* 117 572–586. 10.1111/jam.12545 24833550

[B12] Cole L. K. Kwochka K. W. Kowalski J. J. Hillier A. (1998). Microbial flora and antimicrobial susceptibility patterns of isolated pathogens from the horizontal ear canal and middle ear in dogs with otitis media. *J. Am. Vet. Med. Assoc.* 212 534–538. 9491161

[B13] Delgado M. Neto I. Correia J. H. D. Pomba C. (2007). Antimicrobial resistance and evaluation of susceptibility testing among pathogenic enterococci isolated from dogs and cats. *Int. J. Antimicrob. Agents* 30 98–100. 10.1016/j.ijantimicag.2007.03.007 17509838

[B14] Dos Santos T. P. Damborg P. Moodley A. Guardabassi L. (2016). Systematic review on global epidemiology of methicillin-resistant staphylococcus pseudintermedius: inference of population structure from *Multilocus* sequence typing data. *Front. Microbiol.* 7:1599. 10.3389/fmicb.2016.01599 27803691 PMC5067483

[B15] Dowling P. M. (1996). Antimicrobial therapy of skin and ear infections. *Can. Vet. J.* 37 695–699. 8939340 PMC1576525

[B16] ESTUDIO EPINE-EPPS (2017). EPINE-Point prevalence survey of healthcare-associated infections and antimicrobial use in acute care hospitals, ECDC, 2016-2017. Available online at: https://hws.vhebron.net/epine/Global/EPINE-EPPS%202017%20Informe%20Global%20de%20Espa%C3%B1a%20Resumen.pdf (accessed December 18, 2020).

[B17] Faires M. C. Tater K. C. Weese J. S. (2009). An investigation of methicillin-resistant staphylococcus aureus colonization in people and pets in the same household with an infected person or infected pet. *J. Am. Vet. Med. Assoc.* 235 540–543. 10.2460/javma.235.5.540 19719444

[B18] Fey P. D. Safranek T. J. Rupp M. E. Dunne E. F. Ribot E. Iwen P. C. (2002). Ceftriaxone-resistant *Salmonella* infection acquired by a child from cattle. *N. Engl. J. Med.* 342 1242–1249. 10.1056/nejm200004273421703 10781620

[B19] Frank L. A. Kania S. A. Kirzeder E. M. Eberlein L. C. Bemis D. A. (2009). Risk of colonization or gene transfer to owners of dogs with meticillin-resistant Staphylococcus pseudintermedius. *Vet. Dermatol.* 20 496–501. 10.1111/j.1365-3164.2009.00826.x 20178487

[B20] Guardabassi L. Schwarz S. Lloyd D. H. (2004). Pet animals as reservoirs of antimicrobial-resistant bacteria. *J. Antimicrob. Chemother.* 66 700–709. 10.1093/jac/dkh332 15254022

[B21] Hanselman B. A. Kruth S. A. Rousseau J. Weese J. S. (2009). Coagulase positive staphylococcal colonization of humans and their household pets. *Can. Vet. J.* 50 954–958. 19949556 PMC2726022

[B22] Hariharan H. Coles M. Poole D. Lund L. Page R. (2006). Update on antimicrobial susceptibilities of bacterial isolates from canine and feline otitis externa. *Can. Vet. J.* 47 253–255. 16604982 PMC1371054

[B23] Jackson C. R. Fedorka-Cray P. J. Davis J. A. Barrett J. B. Frye J. G. (2009). Prevalence, species distribution and antimicrobial resistance of enterococci isolated from dogs and cats in the United States. *J. Appl. Microbiol.* 107 1269–1278. 10.1111/j.1365-2672.2009.04310.x 19486402

[B24] Jung W. K. Shin S. Park Y. K. Lim S. K. Moon D. C. Park K. T. (2020). Distribution and antimicrobial resistance profiles of bacterial species in stray cats, hospital-admitted cats, and veterinary staff in South Korea. *BMC Vet. Res.* 16:109. 10.1186/s12917-020-02326-2 32272916 PMC7147017

[B25] Kawakami T. Shibata S. Murayama N. Nagata M. Nishifuji K. Iwasaki T. (2010). Antimicrobial susceptibility and methicillin resistance in staphylococcus *Pseudintermedius* and *Staphylococcus schleiferi* subsp. coagulans isolated from dogs with pyoderma in Japan. *J. Vet. Med. Sci.* 72 1615–1619. 10.1292/jvms.10-0172 20703027

[B26] Kroemer S. El Garch F. Galland D. Petit J. L. Woehrle F. Boulouis H. J. (2014). Antibiotic susceptibility of bacteria isolated from infections in cats and dogs throughout Europe (2002-2009). *Comp. Immunol. Microbiol. Infect. Dis.* 37 97–108. 10.1016/j.cimid.2013.10.001 24447508

[B27] Lappin M. R. Blondeau J. Boothe D. Breitschwerdt E. B. Guardabassi L. Lloyd D. H. (2017). Antimicrobial use guidelines for treatment of respiratory tract disease in dogs and cats: antimicrobial guidelines working group of the International society for companion animal infectious diseases. *J. Vet. Intern. Med.* 31 279–294. 10.1111/jvim.14627 28185306 PMC5354050

[B28] Lloyd D. H. (2007). Reservoirs of antimicrobial resistance in pet animals. *Clin. Infect. Dis.* 45(Suppl. 2) S148–S152. 10.1086/519254 17683019

[B29] McEwen S. A. Fedorka-Cray P. J. (2017). Antimicrobial use and resistance in animals. *Lancet Planet Heal* 34(Suppl. 3) S93–S106. 10.1016/S2542-5196(17)30142-0 11988879

[B30] Nigg A. Brilhante M. Dazio V. Clément M. Collaud A. Gobeli Brawand S. (2019). Shedding of OXA-181 carbapenemase-producing *Escherichia coli* from companion animals after hospitalisation in Switzerland: an outbreak in 2018. *Euro Surveill.* 24 1900071. 10.2807/1560-7917.ES.2019.24.39.1900071 31576806 PMC6774230

[B31] Pantosti A. (2012). Methicillin-resistant *Staphylococcus aureus* associated with animals and its relevance to human health. *Front. Microbiol.* 3:127. 10.3389/fmicb.2012.00127 22509176 PMC3321498

[B32] Pedersen K. Pedersen K. Jensen H. Finster K. Jensen V. F. Heuer O. E. (2007). Occurrence of antimicrobial resistance in bacteria from diagnostic samples from dogs. *J. Antimicrob. Chemother.* 60 775–781. 10.1093/jac/dkm269 17644533

[B33] Petersen A. D. Walker R. D. Bowman M. M. Schott H. C. Rosser E. J. (2002). Frequency of isolation and antimicrobial susceptibility patterns of *Staphylococcus* intermedius and *Pseudomonas aeruginosa* isolates from canine skin and ear samples over a 6-year period (1992-1997). *J. Am. Anim. Hosp. Assoc.* 38 407–413. 10.5326/0380407 12220023

[B34] Prescott J. F. Brad Hanna W. J. Reid-Smith R. Drost K. (2002). Antimicrobial drug use and resistance in dogs. *Can. Vet. J.* 43 107–116. 10.1111/j.1751-0813.2002.tb11003.x 11842592 PMC339174

[B35] Qekwana D. N. Oguttu J. W. Sithole F. Odoi A. (2017). Burden and predictors of Staphylococcus aureus and S. pseudintermedius infections among dogs presented at an academic veterinary hospital in South Africa (2007-2012). *PeerJ* 5 e3198. 10.7717/peerj.3198 28417060 PMC5392248

[B36] Smith D. L. Harris A. D. Johnson J. A. Silbergeld E. K. Morris J. G. (2002). Animal antibiotic use has an early but important impact on the emergence of antibiotic resistance in human commensal bacteria. *Proc. Natl. Acad. Sci. U.S.A.* 99 6434–6439. 10.1073/pnas.082188899 11972035 PMC122966

[B37] Weese J. S. Blondeau J. Boothe D. Guardabassi L. G. Gumley N. Papich M. (2019). International society for companion animal infectious diseases (ISCAID) guidelines for the diagnosis and management of bacterial urinary tract infections in dogs and cats. *Vet. J.* 247 8–25. 10.1016/j.tvjl.2019.02.008 30971357

[B38] Weese J. S. Blondeau J. M. Boothe D. Breitschwerdt E. B. Guardabassi L. Hillier A. (2011). Antimicrobial use guidelines for treatment of urinary tract disease in dogs and cats: antimicrobial guidelines working group of the international society for companion animal infectious diseases. *Vet. Med. Int.* 2011 263768. 10.4061/2011/263768 21776346 PMC3134992

[B39] White D. G. Zhao S. Sudler R. Ayers S. Friedman S. Chen S. (2002). The isolation of antibiotic-resistant *Salmonella* from retail ground meats. *N. Engl. J. Med.* 345 1147–1154. 10.1056/nejmoa010315 11642230

[B40] Windahl U. Bengtsson B. Nyman A. K. Holst B. S. (2015). The distribution of pathogens and their antimicrobial susceptibility patterns among canine surgical wound infections in Sweden in relation to different risk factors. *Acta Vet. Scand.* 57 11. 10.1186/s13028-015-0102-6 25886937 PMC4361205

[B41] Witte W. (1998). Medical consequences of antibiotics use in agriculture. *Science* 279 996–997. 10.1126/science.279.5353.996 9490487

